# Artemisinin and Its Derivatives as Potential Anticancer Agents

**DOI:** 10.3390/molecules29163886

**Published:** 2024-08-16

**Authors:** Luan Wen, Ben Chung-Lap Chan, Ming-Hua Qiu, Ping-Chung Leung, Chun-Kwok Wong

**Affiliations:** 1 Institute of Chinese Medicine and State Key Laboratory of Research on Bioactivities and Clinical Applications of Medicinal Plants, The Chinese University of Hong Kong, Hong Kong, China; 1155209219@link.cuhk.edu.hk (L.W.); pingcleung@cuhk.edu.hk (P.-C.L.); ck-wong@cuhk.edu.hk (C.-K.W.); 2 State Key Laboratory of Phytochemistry and Plant Resources in West China, Yunnan Key Laboratory of Natural Medicinal Chemistry, Kunming Institute of Botany, Chinese Academy of Sciences, Kunming 650201, China; mhchiu@mail.kib.ac.cn; 3 Department of Chemical Pathology, Faculty of Medicine, The Chinese University of Hong Kong, Hong Kong, China; 4 Li Dak Sum Yip Yio Chin R & D Centre for Chinese Medicine, The Chinese University of Hong Kong, Hong Kong, China

**Keywords:** artemisinin, dihydroartemisinin, artesunate, anticancer activity

## Abstract

Artemisinin is a natural sesquiterpene lactone obtained from the traditional Chinese medicinal herb *Artemisia annua* L. (*qinghao*). Artemisinin and its derivatives share an unusual endoperoxide bridge and are extensively used for malaria treatment worldwide. In addition to antimalarial activities, artemisinin and its derivatives have been reported to exhibit promising anticancer effects in recent decades. In this review, we focused on the research progress of artemisinin and its derivatives with potential anticancer activities. The pharmacological effects, potential mechanisms, and clinical trials in cancer therapy of artemisinin and its derivatives were discussed. This review may facilitate the future exploration of artemisinin and its derivatives as effective anticancer agents.

## 1. Introduction

Cancer is the second most deadly disease in the world after cardiovascular disease and seriously threatens human health and life [1]. Multiple approaches are applied for cancer treatment, including surgery, radiation therapy, chemotherapy, and immunotherapy. However, the effects of existing therapies are still limited, especially for those patients with advanced tumors with brain and liver metastases. Therefore, developing effective cancer treatments remains a great focus of scientific research [2]. Natural products, especially compounds with traditional medicinal values, inspire antitumor drug development, as more than half of the approved antitumor drugs are natural products or their derivatives. Mining potential anticancer agents from natural products is conducive to cancer chemotherapy development [3].

Artemisinin is a natural sesquiterpene lactone obtained from *Artemisia annua* L., which has been used as a traditional fever remedy for more than two thousand years in China. Since Chinese scientists isolated and identified artemisinin from *A. annua* in the 1970s, artemisinin has become a widely used antimalarial drug worldwide [4]. However, the bioactivity of artemisinin is limited because of its low solubility and poor bioavailability. To overcome these disadvantages, some artemisinin derivatives were synthesized, such as dihydroartemisinin, artesunate, artemether, arteether, and so on. These derivatives exhibit improved pharmacokinetics, and some of them possess effective clinical effects [5].

With the deepening of research on artemisinin and its derivatives, other biological activities besides anti-malaria effects have also been reported. Notably, artemisinin and its derivatives have exhibited anticancer activity against various cancer cell lines and animal tumor models, and several clinical trials have proven their potential as anticancer agents [5]. In this article, we provide a comprehensive overview of artemisinin and its derivatives as anticancer agents. We describe the chemical features of artemisinin and its derivatives and summarize the reported pharmacological effects and potential mechanisms in the past five years, while the clinical applications of artemisinin and its derivatives in cancer therapy are discussed.

## 2. Chemical Features of Artemisinin and Its Representative Derivatives

**Figure 1:**
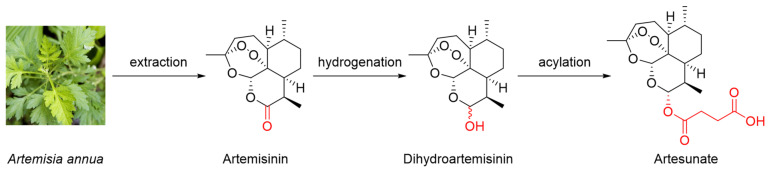
Chemical structures of artemisinin and its typical derivatives.

Artemisinin is a sesquiterpene lactone characterized by a special peroxide group (Figure 1), which plays an important role in its antimalarial and anticancer effects [6]. Because of the limited content of artemisinin in *A. annua*, obtaining large quantities of artemisinin efficiently and economically to meet the great demand remains challenging. The extraction efficiency is improved by modified methods, such as Soxhlet, ultrasound- and microwave-assisted, and supercritical fluid extraction [7]. As for chemosynthesis, because of the high cost and low yield, it is hard to chemically synthesize artemisinin for commercial production. Nowadays, with the development of molecular biology, biosynthesis has attracted increasing attention in artemisinin preparation rather than directly extracting artemisinin from *A. annua* [8].

Dihydroartemisinin is a typical semisynthetic derivative of artemisinin with the lactone ring modified into a hydroxyl group through mild hydrogenation (Figure 1) [9]. Furthermore, artesunate is another commonly used artemisinin derivative that is obtained by the acylation of dihydroartemisinin with succinic anhydride under basic conditions (Figure 1) [10]. Because of the introduction of hydrophilic groups in the modification process, the solubilities of dihydroartemisinin and artesunate are improved. Furthermore, pharmacokinetics analysis has suggested that artesunate can be rapidly hydrolyzed into dihydroartemisinin under physiological conditions [11]. In a clinical trial, the apparent elimination clearance of dihydroartemisinin increased by 24.9% with the increasing treatment time of artesunate, indicating the autoinduction of metabolism [12]. Dihydroartemisinin and artesunate are the most representative derivatives of artemisinin and are commonly used as substitutes for artemisinin.

## 3. Anticancer Effects of Artemisinin and Its Derivatives

Since the anticancer effects of artemisinin were reported in 1995, numerous studies have found that artemisinin and its derivatives exhibit impressive anticancer effects against various types of cancers, including leukemia, glioma, melanoma, colorectal cancer, breast cancer, ovarian cancer, prostate cancer, renal cancer, gastric cancer, etc. [13]. In the past five years, abundant studies have reported various anticancer activities of artemisinin and its representative monomeric derivatives (especially dihydroartemisinin and artesunate), while some of the other synthesized derivatives have also exhibited potent anticancer effects. Meanwhile, artemisinin-derived dimers and trimers have attracted increasing attention because some of them exhibit more potent anticancer activities than monomeric derivatives [5]. In addition, combination treatments as well as nanomedicine are under study to improve the anticancer effects of artemisinin and its derivatives [13].

### 3.1. Anticancer Effects of Artemisinin and Its Derived Monomers

#### 3.1.1. Anticancer Effects on Breast Cancer

Breast cancer has become the most frequent cancer in women globally and is the leading lethal cancer among females [14]. Even though many early stage breast cancers can be cured due to advancements in diagnostic and therapeutic technology, the high rate of metastasis and recurrence leads to a bleak outlook for breast cancer prognosis [15]. Artemisinin and its derivatives could be a potential treatment for this public health situation, as previous studies have reported that artemisinin and its derived monomers show anticancer effects against breast cancer in various ways. 

**Figure 2:**
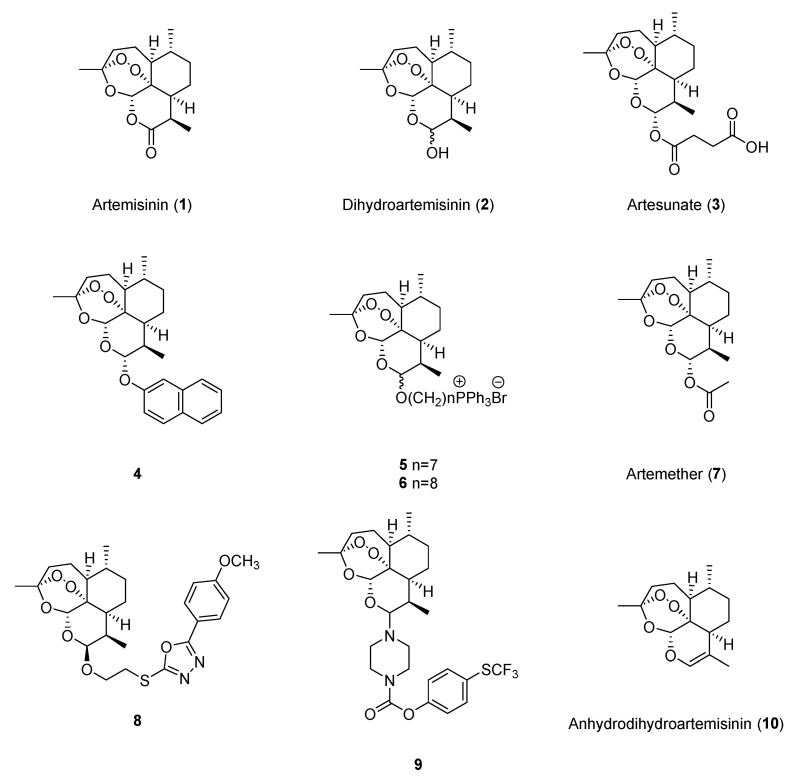
Chemical structures of artemisinin and its monomeric derivatives.

Artemisinin (**1**, Figure 2) could suppress cell growth [16], reduce angiogenesis-related factors [17], and induce ferroptosis [18] in breast cancer cell lines. Dihydroartemisinin (**2**, Figure 2) exhibited anticancer effects against breast cancer by suppressing cell proliferation [16], inhibiting angiogenesis [19], inducing autophagy [20] and pyroptosis [21], and targeting cancer stem cells (CSCs) [20]. Dihydroartemisinin is more potent than artemisinin, as the IC_50_ values at 24 h were lower on MCF-7 (129.1 μM versus 396.6 μM) and MDA-MB-231 (62.95 μM versus 336.63 μM) [16]. Additionally, artesunate (**3**, Figure 2) was reported to induce the apoptosis of breast cancer cells, and the IC_50_ values at 24 h against MCF-7 and 4T1 cells were 83.28 µM and 52.41 μM, respectively [22].

#### 3.1.2. Anticancer Effects on Lung Cancer

Lung cancer is the first leading cause of cancer death worldwide, and finding novel treatments for lung cancer, including drugs as well as therapeutic methods, is urgently needed. As seen in the previous five years of studies, artemisinin and its derivatives have shown significant anticancer effects against lung cancer.

Artemisinin could inhibit cell proliferation and metastasis [23], as well as induce apoptosis [24], to exhibit anticancer effects against lung cancer. The IC_50_ values of artemisinin on A549 cells and H1299 cells were 28.8 μg/mL and 27.2 μg/mL, respectively [24]. Dihydroartemisinin showed anticancer effects by inducing ferroptosis and apoptosis [25,26], inactivating signal transducer and activator of transcription 3 (STAT3) [27], suppressing aerobic glycolysis [28], and modulating the immune response [29,30]. The IC_50_ values at 48 h of dihydroartemisinin on PC9 and NCI-H1975 cells were respectively 19.68 μM and 7.08 μM [27]. Artesunate was also reported to exert anticancer activities against lung cancer through inducing ferroptosis [26] and inhibiting aerobic glycolysis [28]. Moreover, an artemisinin derivative with a naphthalene unit, **4** (Figure 2), was reported to have antiproliferation effects against H1299 (IC_50_ = 0.09 μM) and A549 cells (IC_50_ = 0.44 μM), and could induce ferroptosis in H1299 cells [31]. Additionally, two synthesized artemisinin ester derivatives, **5** and **6** (Figure 2), exhibited potent anticancer effects against A549 cells, and the values of IC_50_ were 126.3 nM and 138.0 nM, respectively [32].

Furthermore, in 2021, Hill et al. revealed that the mutation of kelch-like ECH-associated protein 1 (KEAP1) could participate in the sensitivity of non-small cell lung cancer (NSCLC) cell lines to artesunate through siRNA and small-molecule inhibitor studies, indicating that the combination of artesunate and nuclear factor erythroid 2-related factor 2 (NRF2) inhibitors could become a novel treatment for patients with NSCLC, especially for those with KEAP1/NRF2 pathway mutations [33].

#### 3.1.3. Anticancer Effects on Liver Cancer

As a prevalent cancer of the digestive system, hepatocellular carcinoma (HCC) has a high morbidity rate (4.2%) and fatality rate (7.8%), and it is vital to find new drugs for liver cancer treatment, as many diagnosed patients are in the advanced stage, while the treatment effects are limited [34]. Artemisinin, dihydroartemisinin, artesunate, and artemether (**7**, Figure 2) showed potential anticancer effects against liver cancer by inhibiting cell growth and migration [35,36,37], as well as promoting cell death, including apoptosis [38] and ferroptosis [39]. The IC_50_ values at 24 h of dihydroartemisinin in Hep3B, Huh7, PLC/PRF/5, and HepG2 cells were 29.4 μM, 32.1 μM, 22.4 μM, and 40.2 μM, respectively [39]. In 2023, Tran et al. synthesized eight dihydroartemisinin derivatives with 2-mercapto-1,3,4-oxadiazoles, and compound **8** exhibited the best cytotoxic activity against HepG2 and LU-1 cells, while the IC_50_ values were 3.49 μM and 2.22 μM, respectively [40].

Additionally, dihydroartemisinin was proven to have the ability to reduce the expression of yes-associated protein 1 (YAP1), which has been commonly used as a prognostic marker in liver cancer. Further investigations found that the reduction in YAP1 expression could be positively related to the suppression of lipid droplet deposition [41] and interleukin (IL)-18 expression [42]. However, previous research found that artesunate treatment (more than 5 μM) for 24 h exhibited detrimental action on human normal liver cells (BRL-3A and AML12) and induced G0/G1 cell cycle arrest and apoptosis by promoting oxygen species (ROS) accumulation. This study suggested that the possible side effects of artesunate should be considered for cancer therapy [43].

#### 3.1.4. Anticancer Effects on Colorectal Cancer

Colorectal cancer is the third most common and the second most lethal cancer in the world, and drug development has always been under concern. In the past five years, artemisinin and its derivatives were reported to show anticancer effects against colorectal cancer. Artemisinin exhibited anticancer activities by inhibiting cell proliferation and migration [44,45,46], as well as increasing CD8+ T cell infiltration [47]. Dihydroartemisinin was reported to show anticancer effects through inhibiting CSCs [48]. Artesunate treatment could induce cell senescence and autophagy in both SW480 and HCT116 cells (1, 2, 4 μM for 72 h) and CT26 tumor-bearing mice (30 or 60 mg/kg for 24 days) [49]. Compared with artemisinin, dihydroartemisinin and artesunate had better cytotoxic effects on colon cancer cells because COLO 205, HCT116, and DLD-1 cells were sensitive to both dihydroartemisinin and artesunate at a concentration of 100 μM but resistant to artemisinin treatment [50]. In 2021, Lan et al. synthesized 26 artemisinin derivatives and found that compound **9** showed the most effective activity against HCT116 cells (IC_50_ = 0.12 μM) by inhibiting migration, inducing cell cycle arrest, and promoting apoptosis [51].

Hypoxia is an important factor to be considered in cancer treatment, as it participates in resistance to chemotherapy as well as radiotherapy against cancer. To investigate the anticancer effects of dihydroartemisinin in hypoxia, Bader et al. utilized HCT116 Bax−/−Baksh cells, which exhibit intrinsic apoptosis dysfunction, and used HCT116 wild-type cells for comparison. In HCT116 wild-type cells, dihydroartemisinin treatment quickly induced apoptosis in normoxia and promoted non-apoptosis cell death in hypoxia. In HCT116 Bax−/−Baksh cells, dihydroartemisinin induced cell cycle arrest both in normoxia and hypoxia and promoted delayed cell death in hypoxia but showed no significant cell toxicity in normoxia. These results suggested that Bax/Bak expression plays an important role in the anticancer effects of dihydroartemisinin against colon cancer cells in normoxia but does not participate in cell death in hypoxia [52].

Artemisinin and its derivatives are considered to interact with multiple protein targets, and evidence shows that these targets could be unique to specific cells or tissues [53]. In 2024, Geng et al. utilized photoaffinity probes to find specific targets that participate in the anticancer effects of artemisinin against colon cancer. The investigation found that artemisinin specifically inhibited microsomal prostaglandin synthase-2 reversibly, decreased the level of cellular prostaglandin E2, and inhibited cell growth [53]. In 2022, Gong et al. found that the combination of artesunate and a Wnt pathway inhibitor, WNT974, could target KRAS degradation [54]. Additionally, prohibitin 2 and RCHY1 were reported to be targets for the anticancer effects of dihydroartemisinin [55].

Although many studies have reported the anticancer effects of artemisinin and dihydroartemisinin, the conditions set up in the laboratory are very different from the physiological environment of the human body. To investigate the real cytotoxic effect of artemisinin and dihydroartemisinin on colon cancer cells (SW480 and SW620), Otto-Ślusarczyk et al. conducted a study with pharmacologically achievable drug concentrations (1–8 μM), while linoleic acid (LA20, 40 μM) and holotransferrin (TRFi, 50 μM) at physiological levels were added in the culture medium. Results showed that the updated cell culture setting could significantly improve the anticancer effects of artemisinin and dihydroartemisinin, including the induction of apoptosis and IL-6 stimulation [56].

#### 3.1.5. Anticancer Effects on Melanoma

Melanoma is a very aggressive type of skin cancer with a high mortality rate, accounting for 80% of skin cancer-related death cases. There are few effective treatments for melanoma in addition to surgery [57]. As one of the strategies of melanoma immunotherapy, the application of immune checkpoint inhibitors, in particular, antibodies of programmed cell death-1 (PD-1) or programmed cell death-ligand 1 (PD-L1), are widely used to enhance the immune response and induce cancer attack, but they only exhibit effectiveness on a minority of patients [58]. Recent studies highlighted the potential anticancer effects of artemisinin and its derivatives against melanoma through targeting immunomodulation, as artemisinin and dihydroartemisinin could modulate the tumor-infiltrating lymphocytes and improve the immunotherapy effects targeting PD-L1 [58,59,60]. Meanwhile, STAT3 signaling is considered an anticancer target of dihydroartemisinin and artesunate [57,60].

#### 3.1.6. Anticancer Effects on Esophageal Cancer

Esophageal cancer is a type of complicated gastrointestinal malignancy and possesses a poor survival rate because of its aggressiveness. Previous studies have reported that artemisinin inhibited the development of esophageal cancer by inhibiting glycolysis [61]. Additionally, dihydroartemisinin showed anticancer effects against esophageal cancer by inhibiting cell migration, inducing autophagy [62] and cell cycle arrest [63,64], and targeting AKT1, p70S6K [65], and human telomerase reverse transcriptase [66].

#### 3.1.7. Anticancer Effects on Other Cancers

In addition to the above cancer types, artemisinin and its derivatives have been reported to possess anticancer effects against other cancers, including bladder cancer, cervical cancer, gastric cancer, glioma, head and neck cancer, ovarian cancer, etc. (Table 1). Reported effects include inhibiting cell proliferation, migration, and invasion; suppressing angiogenesis and anaerobic glycolysis; and inducing cell cycle arrest and cell death.

### 3.2. Anticancer Effects of Artemisinin-Derived Dimers

In addition to monomers (such as dihydroartemisinin, artesunate, and artemether), dimers and trimers are also included in the derivatives of artemisinin, and some of them exhibited ideal anticancer effects in previous studies [5].

To obtain access to more effective anticancer compounds than artemisinin, the chemical connection of artemisinin-derived precursors such as dihydroartemisinin and artesunate with other anticancer pharmacophores (coumarins, steroids, terpenes, flavonoids, cinnamic acids, etc.) to obtain hybrid compounds is commonly reported. As the anticancer effects of artemisinin-derived hybrids have been well summarized in recent reviews [5,6,90], we have focused on the artemisinin-derived dimers synthesized from two artemisinin-derived groups without other types of pharmacophores in this article, and the anticancer activity studies of artemisinin-derived dimers in the past five years have been reviewed.

**Figure 3:**
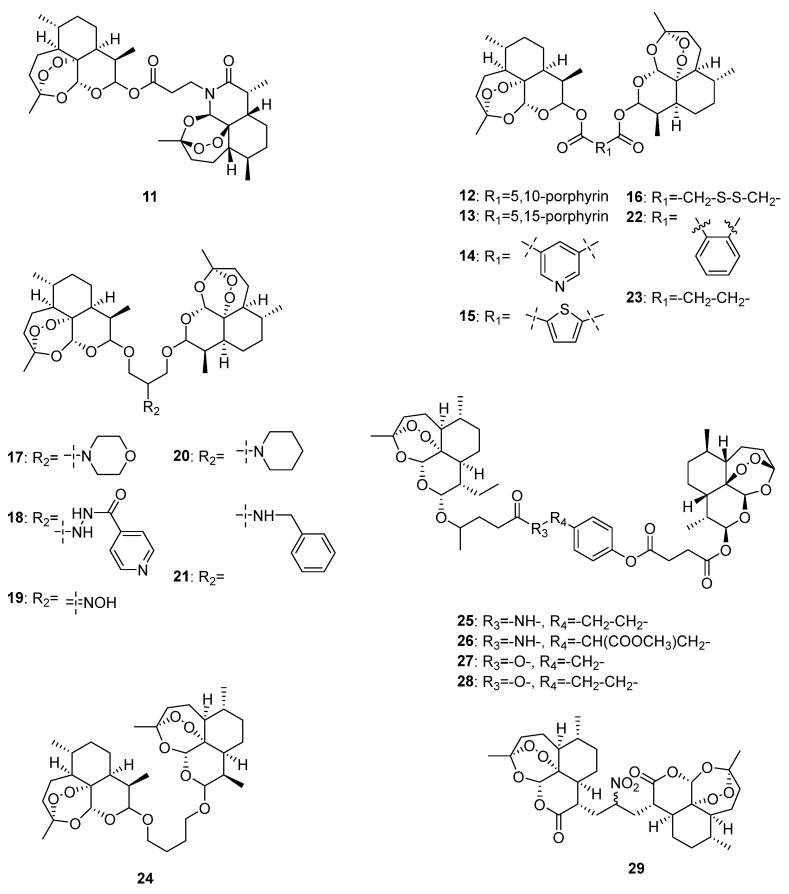
Chemical structures of artemisinin-derived dimers.

In 2024, Zhong et al. designed and synthesized five artemisinin-derived dimers (**11**–**15**, Figure 3) and determined their in vitro anti-proliferative activities. Results showed that compared to artemisinin and dihydroartemisinin, **11**, **14**, and **15** had better anticancer effects against four human tumor cell lines (MCF-7, HepG-2, HCT-116, and BGC-823), while the IC_50_ value of compound **15** was 8.30 μM against human gastric cancer cell line BGC-823. This study indicated that the linker plays an important role in the anticancer activities of artemisinin-derived dimers. It might be helpful to use linkers with small backbones or high polarity to improve the anticancer effects [91].

Nowadays, nanomedicine delivery systems are commonly studied for artemisinin dimer derivatives in cancer treatment to overcome the inherent defects of traditional delivery methods, such as inadequate solubility, broad distribution, and rapid elimination. In 2021, Li et al. designed and synthesized a dihydroartemisinin dimeric prodrug bridged with a disulfide bond linkage (**16**, Figure 3) that could self-assemble into nanoparticles. Further activity determination revealed preferable anticancer effects of **16** compared with those of free dihydroartemisinin, and it could significantly induce apoptosis and suppress aerobic glycolysis in human liver HepG2 cells [92].

In 2020, Elhassanny et al. investigated the anticancer effects of five dihydroartemisinin dimers (**17**–**21**, Figure 3) and found that the oxime dimer NSC735847 (**19**) exhibited selectively cytotoxic effects on two human colon cell lines (HT29 and HCT116) over 24 h treatment, and the values of IC_50_ were 10.95 and 11.85 μM, respectively. Further mechanistic investigations showed that NSC735847 was activated by heme and induced cell death through the endoplasmic reticulum stress pathway [93].

In 2021, Botta et al. synthesized seven dimers of dihyartemisinin and artesunate (**22**–**28**, Figure 3) and determined their anticancer effects on human metastatic melanoma RPMI7951 cells. Among the seven dimers, **27** exhibited the best anticancer activity selectively towards melanoma (IC_50_ = 0.05 μM). According to the effect comparison of **27** and **28**, a methylene group plays an important role in the anticancer effects of artesunate dimers [94].

Unlike the above artemisinin-derived dimers connected by oxygen atoms, Kalen et al. synthesized a novel artemisinin-derived carba-dimer **29** (Figure 3) through a selenoxide elimination method. Anticancer effect determination showed that **29** had better anti-proliferative effects on human head–neck (Cal27), breast (MDA-MB-231), prostate (PC-3), and melanoma (A375 and MD-435) cancer cell lines than artemisinin, and it could lead to oxidative stress and cell cycle G1 arrest of cancer cells [95].

Although artemisinin derivatives have demonstrated promising anticancer activity, few studies have been conducted on their configuration–activity relationship. To address this, Yue et al. systematically synthesized 11 artemisinin-derived dimers with different stereogenic centers and evaluated their anticancer effects. Results showed that the dimers with β, β and α, β configurations exhibited better inhibitory effects on MCF-7 and HepG2 cancer cells than those with the α, α configuration [96].

### 3.3. Combination Therapy with Artemisinin and Its Derivatives against Cancer

Compared with those of monotherapy, evidence highlighted the significantly improved anticancer effects of combination therapy with artemisinin and its derivatives. The combination therapy mainly includes drug–drug and chemical–physical combination therapy [97].

The application of combination treatment using artemisinin and its derivatives with commonly used chemotherapy drugs, such as cisplatin, carboplatin, doxorubicin, temozolomide, etc., always exhibits significantly improved anticancer effects [98]. For example, compared with the 48 h treatment with artesunate (9 μg/mL) or carboplatin (7 μg/mL) alone, the combination treatment exhibited stronger anticancer effects on A549 and H1299 cells [24]. The combination of formononetin and dihydroartemisinin showed synergistic anticancer effects on U937 and KG-1 cells through inducing cell cycle arrest and apoptosis [99]. On the other hand, combination treatment could reverse drug resistance in cancer therapy. For example, artesunate (2.5–50 μM) could dose dependently increase the inhibitory effects against two doxorubicin-resistant (A549/TAX and A549/DDP) cell lines [100].

In addition to chemotherapy alone, chemical–physical combination therapy is also one of the current research hotspots for improving anticancer effects. The commonly studied physical treatments include conventional radiation therapy and emerging photodynamic therapy [101]. For example, artesunate could enhance radiosensitivity in the human esophageal cancer cell line TE1 by inducing cell apoptosis, reversing G2/M cell cycle arrest, and delaying DNA repair [102], and dihydroartemisinin (30 μM, 24 h) could enhance the effect of 5-aminolevulinic acid-mediated photodynamic therapy on HeLa cells [103].

### 3.4. Nanomedicine in Anticancer Therapy Using Artemisinin and Its Derivatives

Notably, in addition to traditional drug delivery methods, a rising number of innovative nanomedicine-based delivery systems have been studied for anticancer treatment using artemisinin and its derivatives in recent years. The application of nanomedicines has been considered an effective strategy for the accurate delivery of chemotherapeutic agents, therefore reducing drug resistance and systemic effects [97].

For example, Patil et al. developed a type of artesunate-loaded, layer-by-layer (LBL)-coated, solid lipid nanoparticle (SLN)-loaded microneedles for breast cancer treatment through the transpapillary route. According to the results of the cell line study and ex vivo release study, compared to artesunate, the LBL-coated, SLN-loaded microneedles exhibited less proliferation of cell line MCF-7 (7.92 ± 1.54%) and higher ex vivo release (84.75 ± 2.02%), indicating that they could be an effective alternative delivery method for artesunate against breast cancer [104].

In 2024, Cui et al. designed and prepared Fe_3_O_4_ nanoparticle micelles loaded with artemisinin. Because of the cell ferroptosis induced by improving ROS levels, the cancer cell suppression rate of the micelles was up to 85%, which was much more effective than free artemisinin [18]. To improve the tumor targeting of dihydroartemisinin, Shen et al. prepared novel alkyl glucoside-modified dihydroartemisinin liposomes that could precisely target glucose transporter 1 (GLUT1). With the glucose segment as the targeting head, the targeting ability of the liposomes to GLUT1 was significantly improved both in human liver HepG2 cells and in H22 tumor-bearing mice [105].

## 4. Anticancer Mechanisms of Artemisinin and Its Derivatives

Artemisinin and its derivatives have been shown to possess potent anticancer effects, including impeding cancer proliferation and metastasis, inducing cell cycle arrest, and inhibiting angiogenesis. These effects are mediated by changes in multiple signaling pathways, interfering with various hallmarks of cancer [106]. Furthermore, artemisinin and its derivatives also target cell death induction, which was mostly considered caspase-dependent and mitochondrial pathway-mediated apoptosis in previous studies. Recently, more and more evidence has shown that artemisinin and its derivatives exhibit anticancer effects through nonapoptotic pathways, such as ferroptosis and autophagy [98]. Additionally, recent studies have also suggested new potential mechanisms for the anticancer effects of artemisinin and its derivatives, including immune regulation, aerobic glycolysis inhibition, STAT3 targeting, and CSC inhibition [5]. In this chapter, we summarize the reported underlying mechanism investigations of the anticancer effects of artemisinin and its derivatives in the past five years.

### 4.1. Cancer Proliferation and Metastasis

The anticancer activity of artemisinin and its derivatives involves multiple targets and pathways. The inhibition of cancer cell proliferation, invasion, and metastasis is the most studied phenotype, and the mechanisms have been well studied [106].

Artemisinin exerted its anticancer effects on human HCC cell lines (HepG2 and Hep3B) by inhibiting Forkhead box M1 [36]. In colon cancer, artemisinin exhibited anti-proliferation effects by inhibiting the expression of miR-22 and CyclinD1 [44], and when combined with 5-fluorouracil, artemisinin improved its effects on inhibiting proliferation and migration in colon cancer cells via PI3K/Akt signaling [45]. Dihydroartemisinin inhibited the proliferation and metastasis of breast cancer by downregulating the TGF-β1/Smad signaling pathway and CIZ1 expression [16], inhibiting the growth and migration of human lung cancer cells by downregulating the PRIM2/SLC7A11 axis [25], inhibiting fibronectin-1 and integrin-β1 expression via the PI3K-Akt pathway [37], and inhibiting the proliferation, colony formation, and invasiveness of colon cancer cells by inhibiting NRP2, N-cadherin, and Vimentin expression [46].

Artesunate inhibited the proliferation, migration, and invasion of two human NSCLC cell lines (A549 and H1299) by decreasing the expression of HuR and matrix metalloproteinase (MMP)-9 proteins [24], and its anti-migratory activity might also be due to the inhibition of the epidermal interstitial transformation by downregulating BTBD7 mRNA expression [23]. Artemether inhibited the proliferation, invasion, and migration of Hep3B2.1-7 cells by targeting cytochrome P450 family 2 subfamily J member 2 (CYP2J2) [35].

### 4.2. Angiogenesis

In 2020, Dong et al. investigated the in vivo anticancer effects of artemisinin on MDA-MB-231 tumor-bearing mice. ELISA results showed the serum vascular endothelial growth factor (VEGF) and hypoxia-inducible factor 1α levels in the mice group with a high dose of artemisinin were significantly higher than those in the control group (*p* < 0.05). A fluorescence quantitative assay suggested that the artemisinin treatment could reduce the expression of notch signaling-related factors notch1, Dll4, and Jagged1, indicating that artemisinin could inhibit angiogenesis by downregulating the notch signaling pathway to exhibit anticancer effects on breast cancer [17]. Rao et al. also studied the anti-angiogenesis effects of dihydroartemisinin on breast cancer and found that dihydroartemisinin downregulated the expression of VEGF and MMP-2/-9 in human breast MDA-MB-231 cancer cells. According to the results of immunofluorescence and Western blot analyses, the underlying mechanism was concluded to be the inhibition of the PI3K/Akt/NF-κB and ERK/NF-κB signaling pathways [19].

### 4.3. Cell Cycle

Uncontrolled division is one of the main characteristics of cancer cells and an important reason for their rapid proliferation. Blocking the cell cycle of cancer cells is an effective way to inhibit tumor growth. Artemisinin and its derivatives have been reported to exert anticancer effects by causing cell cycle arrest [106].

In 2020, Ma et al. found that dihydroartemisinin significantly induced G2/M cell cycle arrest in human esophageal Eca109 cancer cells by inducing ROS-mediated autophagy, which is related to DNA damage response caused by the TRF2 degradation [63]. In 2022, Xu et al. synthesized a group of artemisinin derivatives with TPP+ moieties that target mitochondria, and one of the derivatives was found to show effective anticancer activities against two human bladder cancer cell lines, J82 and T24, while the IC_50_ values were 61.8 nM and 56.9 nM, respectively. Although the derivative did not induce cancer cell death, it significantly induced G1-phase cell cycle arrest. Further, Western blot analysis showed that D8-T could decrease the expression of cyclin D1, CDK6, and CDK4 and increase the expression of p21. RNA-seq analysis showed consistent results that most observed differentially expressed genes compared to the control were linked to the cell cycle [32].

### 4.4. Cell Death

Resisting cell death is one of the core hallmarks of cancer, and inducing cell death is an important strategy for cancer treatment. In addition to the most studied routes of apoptosis and autophagy, several alternative routes of cell death have been proposed and discussed, including oncosis, ferroptosis, and pyroptosis. Recently, the anticancer effects of artemisinin and its derivatives through inducing cell death, especially ferroptosis, have attracted more and more attention [107].

#### 4.4.1. Apoptosis

Apoptosis is an important form of programmed cell death and is commonly considered caspase dependent and mediated by the mitochondrial pathway. As insufficient apoptosis can lead to the uncontrolled proliferation of cancer cells, which is related to tumor occurrence and drug resistance, inducing apoptosis plays a crucial role in cancer therapy [13].

In 2020, Zhou et al. investigated the apoptosis-inducing effects of the combination of artemisinin derivatives with tumor necrosis factor-related apoptosis-inducing ligand (TRAIL) variants on human colon cancer cells. Results showed that artesunate or dihydroartemisinin significantly increased the cell apoptosis proportion under the treatment of the death receptor 5 (DR5)-specific TRAIL variant DHER in two human colon cancer cell lines (HCT116 and DLD-1). Dihydroartemisinin could induce the sensitivity of DHER for apoptosis induction in HCT116 cells by regulating DR5 expression via P53, and the effect was confirmed in a 3D tumor spheroid model [50].

Human heat shock protein 70 (HSP70) is considered an important protein that can suppress apoptosis both in the extrinsic and intrinsic pathways and participates in increasing drug resistance. In 2020, Pirali et al. purified recombinant HSP70 protein, and a carbonic anhydrase refolding assay showed that artesunate could dose dependently inhibit the HSP70 ATPase activity in vitro. In two breast cancer cell lines (MCF-7 and 4T1), artesunate significantly reduced the expression of HSP70 and another anti-apoptosis protein, Bcl-2, and induced cleaved caspase-9 expression. These results suggested that artesunate could induce caspase-dependent apoptosis by downregulating HSP70 expression [22].

#### 4.4.2. Autophagy

Autophagy, also known as type II programmed cell death, refers to the process of the degradation and recycling of organelles and parts of the cytoplasm in cells. Autophagy is a double-edged sword because it is linked to both the survival and death of cells. At present, it is generally believed that the promotion of autophagy is related to the occurrence and development of tumors [107].

In 2022, Huang et al. found that artesunate induced excessive ROS production to induce senescence and autophagy and eventually lead to cell death in human colon cells (SW480 and HCT116). The mechanism of autophagy promotion is the activation of endoplasmic reticulum stress and unfolded protein response (UPR) through the upregulation of the IRE1α pathway [49]. In 2020, Chen et al. investigated the anticancer effects of dihydroartemisinin on esophageal cancer and found that dihydroartemisinin treatment activated autophagy by inhibiting the Akt/mTOR signaling pathway in two human squamous cell lines (TE-1 and Eca109) [62].

In 2023, Chatterjee et al. investigated the role of C-type lectin-like domain family 12 member A (CLEC12A) in the anticancer effects of artemisinin on non-hematopoietic cancer. In vivo experimental studies showed that artemisinin reduced CLEC12A expression and exhibited anticancer effects on 4T1 tumor-bearing mice through a non-canonical pathway, which was different from that known for toll-like receptor-mediated signaling in leukemia. Western blot analyses revealed that artemisinin reduced the expression of autophagic markers beclin1 and LC3α/β, indicating that artemisinin could dose dependently suppress autophagy and therefore inhibit NF-κB-mediated inflammation [20].

#### 4.4.3. Ferroptosis

Ferroptosis is an iron-dependent programmed cell death process characterized by the accumulation of lipid peroxides. The failure of antioxidant defenses leads to uncontrolled lipid peroxidation and cell death. Because of the regulatory role of ferroptosis activation in the growth of human cancer cells, inducing ferroptosis has become a cancer therapy of great interest in recent years [31].

In 2020, Yuan et al. found that dihydroartemisinin (40 μM and 60 μM) significantly inhibited cell growth and induced ferroptosis in two human lung cancer cell lines (NCI-H23 and XWLC-05) by downregulating the level of PRIM2 after 48 h treatment. Further investigations suggested that both dihydroartemisinin treatment and the loss of PRIM2 could lead to a decreased GSH level and induce cellular lipid ROS and mitochondrial MDA expression. The knockdown of PRIM2 enhanced the sensitivity of NCI-H23 cells to dihydroartemisinin therapy, while the overexpression of PRIM2 had the opposite effect, indicating that dihydroartemisinin induced ferroptosis in lung cancer cells by upregulating the PRIM2/SLC7A11 pathway [25].

In 2021, Wang et al. found that primary liver cancer cells with dihydroartemisinin treatment displayed typical features of ferroptosis. Further studies showed that dihydroartemisinin activated all three branches of UPR and Chac glutathione-specific γ-glutamylcyclotransferase 1 (CHAC1). After the knockdown of activating transcription factor 4/6 or X-box binding protein 1 (XBP1), the induction effect of dihydroartemisinin on cancer cell ferroptosis, as well as the activity of CHAC1, was weakened, indicating that dihydroartemisinin could induce ferroptosis by upregulating CHAC1 via UPR [39].

In 2022, Xie et al. synthesized a series of artemisinin derivatives with different aryl substituents in the C-10 position and obtained an artemisinin derivative, ART1, with significantly improved toxicity against two human NSCLC cell lines (A549 and H1299). Further chemical proteomic approaches found that ART1 could induce ferroptosis by targeting HSD17B4 protein, which is responsible for the selective oxidation of fatty acids [31].

#### 4.4.4. Pyroptosis

In addition to apoptosis, autophagy, and ferroptosis, pyroptosis is another programmed and inflammatory cell death that has a close correlation to different human diseases, especially malignancies. As pyroptosis can not only suppress the occurrence and development of cancer but also increase drug sensitivity, inducing pyroptosis could have great potential in cancer treatment [21].

In 2021, Li et al. found that dihydroartemisinin induced pyroptosis in two human breast cancer cell lines (MCF-7 and MDA-MB-231) and reduced the expression of melanoma 2 (AIM2), caspase-3, and gasdermin E (DFNA5). Further mechanistic investigations showed that dihydroartemisin activated the AIM2/caspase-3/DFNA5 pathway to induce the pyroptosis of breast cancer cells [21].

#### 4.4.5. Oncosis

Oncosis, also known as ischemic cell death, is a type of accidental cell death. Oncosis is characterized by cell swelling caused by ion transporter dysfunction and is related to various cellular intercellular activities, including ROS generation reduction, mitochondrial condensation, calpain and cathepsin activation, lysosomal disruption, and endoplasmic reticulum dilation [107].

In 2021, Jiang et al. found that artesunate induced cell death in human HCC cell lines (Hep3B, SMMC7721, HepG2, and Huh7). Further mechanistic investigations showed that artesunate treatment induced the acidification of lysosomes to activate ferritin degradation and therefore increased the labile iron pool. The iron redistribution induced excessive ROS accumulation in the endoplasmic reticulum and eventually led to cell death [38].

### 4.5. Immunomodulation in Cancer

Apart from conventional chemotherapeutic treatments, immunotherapy is an emerging cancer treatment due to its impressive antitumor effects and clinical benefits. Recently, the potential of artemisinin and its derivatives as immunomodulators has attracted increasing attention [98].

In 2022, Wang et al. investigated the effects of artemisinin and dihydroartemisinin on adaptive immune regulation in colon cancer. Results showed that both artemisinin and dihydroartemisin upregulated the level of IL-8 and inhibited Th1 and Th17 differentiation, while dihydroartemisinin could induce the proliferation of Treg cells. In 2023, Hu et al. found that dihydroartemisinin could reduce the expression of an immune checkpoint molecule, B7-H3, in two human NSCLC cell lines, A549 and HCC827, and increase tumor-infiltrating CD8+ T cells in tumor engraftments in a A549 tumor-bearing mouse model [30]. Sun et al. found that artemisinin significantly suppressed cancer proliferation and metastasis against colon cancer by inhibiting chondroitin sulfate synthase 1 (CHSY1). Artemisinin reduced the expression of Ki-67, PD-L1, and CHSY1 and increased CD8+ tumor-infiltrating T cells [47].

In addition to T-cell differentiation, Xiao et al. investigated tumor-associated macrophage (TAM) polarization in a mouse Lewis lung carcinoma model with dihydroartemisinin treatments, and results showed that dihydroartemisinin could polarize TAMs into M1-like phenotypes in a dose-dependent manner [29]. In 2022, Zhang et al. found that artemisinin treatment significantly polarized M2-like phenotypes towards M1-like phenotypes in myeloid-derived suppressor cells through the PI3K/Akt, mTOR, and MAPK signaling pathways, and the function was verified in both melanoma- and liver tumor-bearing mouse models [58].

### 4.6. Glucose Metabolism

As described by the Warburg effect, most cancer cells produce energy through anaerobic glycolysis, which is different from the citric acid cycle of normal cells. Active glycolysis is beneficial to the proliferation and survival of malignant tumors and enhances the invasion ability of cancer cells. Artemisinin and its derivatives have been reported to exert anticancer effects by affecting the glucose metabolism of cancer cells, especially by inhibiting anaerobic glycolysis [98].

In 2022, Wang et al. found that artemisinin inhibited glycolysis in two human esophageal cancer cell lines (KYSE-150 and KYSE-170) by inhibiting the key glycolysis enzymes hypoxia-inducible factor-1α (HIF-1α) and pyruvate kinase M2. Network pharmacology confirmed that artemisinin could target HIF-1α degradation [61].

Furthermore, Zhang et al. investigated the molecular mechanism of artesunate and dihydroartemisinin in cancer metabolic reprogramming. The results showed that both artesunate and dihydroartemisinin suppressed glycolysis in three human NSCLC cells (NCI-H358, NCI-H1975, and PC9) and a Lewis lung cancer mouse model. Both artesunate and dihydroartemisinin treatments decreased the expression of GLUT1, hexokinase, lactate dehydrogenase (LDHA), p-ERK/ ERK, and c-Myc in vitro and in vivo. Furthermore, the overexpression of c-Myc enhanced aerobic glycolysis in PC9 cells and overcame the reduction effects of dihydroartemisinin and artesunate on GLUT1 and LDHA, suggesting that dihydroartemisinin and artesunate could regulate aerobic glycolysis by downregulating the ERK/c-Myc pathway [28].

### 4.7. STAT3

STAT3 is an important transcription factor of various signaling pathways in human malignant tumors and participates in many processes in cancer occurrence and development, such as cancer cell growth, cell cycle, anti-apoptosis effects, and immune evasion. Although numerous studies have focused on suppressing STAT3 as a potential cancer treatment, it is hard to develop an inhibitor directly targeting STAT3. Recently, artemisinin and its derivatives were reported to have potential as direct STAT3 inhibitors [98].

For the treatment of melanoma, hydroartemisinin could significantly reduce the levels of p-STAT3, p65, and IL-10 induced the p-STAT1 both in vitro and in vivo, indicating that dihydroartemisinin exhibits anticancer effects on melanoma by affecting STAT1/STAT3 pathway [59,60]. Furthermore, 48 h treatment with artesunate reduced p-STAT3 and p-Src expression in human A375 melanoma cells, indicating that artesunate could downregulate the Src/STAT3 signaling pathway in melanoma [57].

In 2024, Li et al. investigated the underlying mechanism of anticancer effects of dihydroartemisinin through the STAT3 signaling pathway. Dihydroartemisinin significantly reduced the expression of receptor tyrosine kinase-like orphan receptor 1 (ROR1), the phosphorylation level of STAT3, and the expression of its downstream target c-Myc in two human NSCLC cell lines (PC9 and NCI-H1975). Furthermore, blocking ROR1 could significantly reduce the expression of p-STAT3 and c-Myc, while the overexpression of ROR1 has the opposite effect, indicating that artesunate could downregulate the STAT3/c-Myc pathway by reducing ROR1 in NSCLC cell lines [27].

### 4.8. Cancer Stem Cells

Cancer stem cells (CSCs) are cancer cells that possess stem cell-like characteristics and can form tumors and lead to cancer metastasis and relapse. Therefore, the targeted inhibition of CSCs is commonly considered an effective method for cancer therapy. Recently, several studies have revealed the potential of artemisinin and its derivatives for exerting inhibitory activity against CSCs [98].

In 2023, Chatterjee et al. found that artemisinin treatment could significantly reduce the expression of CSC makers (Sox2, Oct4, Nanog, and ALDH1A1) in 4T1 tumor-bearing mice, indicating that artemisinin could inhibit CSCs to exhibit anticancer effects on breast cancer, which is similar to normal cancer cells [20]. Similarly, Wang et al. studied the inhibitory effects of dihydroartemisinin on CSCs in human colon cancer cells (HCT116 and SW620), and results showed that dihydroartemisinin treatment could significantly reduce the expression of CSC markers (CD133, CD44, Nanog, c-Myc, and OCT4) by downregulating Akt/mTOR pathway [48].

## 5. Clinical Trials of Artemisinin and Its Derivatives

Although the anticancer effects as well as the underlying mechanisms of artemisinin and its derivatives have been studied in various studies, clinical trials of artemisinin and its derivatives in cancer therapy are still limited. As summarized in Table 2, most previous clinical trials focused on artesunate treatment against metastatic breast cancer. While the effectiveness of artemisinin and its derivatives has been confirmed in clinical anticancer therapy, additional clinical studies are still required for the verification of safety and tolerability. Although no serious and irreversible adverse effects have been reported in clinical trials completed to date, the potential toxicity of artemisinin and its derivatives, especially on the audiological system, remains a concern [108].

## 6. Conclusion and Prospective

As commonly used antimalarial drugs, artemisinin and its derivatives have also shown anticancer activities both in vivo and in vitro. In the past five years, studies have mainly reported the anticancer effects of artemisinin and its derivatives on breast cancer, lung cancer, liver cancer, and colon cancer, including inhibiting cell proliferation and metastasis, suppressing angiogenesis, promoting cell cycle arrest, inducing cell death, inhibiting glycolysis, targeting the STAT3 pathway and CSCs, and regulating immunity. Clinical trials have demonstrated the efficacy and safety of artemisinin and artesunate in cancer therapy.

Because of the pharmacokinetic limitations of artemisinin, more studies have focused on dihydroartemisinin, artesunate, and artemether. In addition to these representative derivatives, more chemical modification studies are needed for more effective anticancer agents, and dimers or trimers are worth studying, as they may have stronger activity than monomeric compounds. Moreover, to improve the efficacy of monotherapy of artemisinin and its derivatives, the development of nanomedicine can help improve the drug delivery efficiency and targeting effect. For the study of the mechanism of the anticancer activity of artemisinin and its derivatives, CSCs, immune regulation, and glycolysis are new directions worthy of attention. In addition, with the development of molecular biology, increasing protein targets have been discovered. These new mechanistic studies will provide a reference for combination therapy using artemisinin and its derivatives with other therapeutic approaches, which is conducive to improving anticancer activity more accurately. Furthermore, based on the anticancer pharmacological activity of artemisinin and its derivatives, more clinical studies are needed to verify their efficacy and safety, especially the long-term treatment tolerability.

In summary, artemisinin and its derivatives can be considered potential anticancer agents; however, there is still a long way to go to improve their clinical application effects.

## Figures and Tables

**Table 1:** Anticancer effects of artemisinin and its monomeric derivatives on other cancers.

Compound	Cancer Type	Anticancer Effect	Mechanism	Ref.
Artemisinin	Prostate cancer	Inhibiting invasion and migration	Downregulating the E2F5/TFPI2/MMP pathway	[67]
	Thyroid cancer	Inhibiting anaerobic glycolysis	Downregulating the XIST/miR-93/HIF-1α pathway	[68]
Dihydroartemisinin	Bladder cancer	Inhibiting invasion and migration	Reducing KDM3A and inducing p21	[69]
	Cervical cancer	Inducing ferroptosis	Inducing ferritinophagy	[70]
		Inducing cell growth, invasion, migration, and apoptosis	Reducing Bcl-2, N-cadherin, and Vimentin; inducing RECK	[71]
	Gastric cancer	Inhibiting cell growth, invasion, and migration; inducing G1 cell cycle arrest	Downregulating the cyclin D1-CDK4-Rb pathway, PCNA, and MMP2	[72]
	Glioma	Inhibiting ferroptosis	Upregulating the TUG1/MAZ/FTH1 pathway	[73]
	Head and neck cancer	Inducing ferroptosis	Inducing ROS production	[74]
		Inducing invasion and migration	Regulating miR-195-5p expression	[75]
	Neuroblastoma	Inhibiting cell growth and inducing apoptosis	Regulating taurine, linoleic acid, phenylalanine metabolism, and tryptophan metabolism	[76]
	Osteosarcoma	Inhibiting cell growth, inducing cell death	Inducing the ROS/Erk1/2 pathway and mitochondrial damage	[77]
		Inhibiting angiogenesis	Reducing Loxl2/VEGFA expression	[78]
	Ovarian cancer	Inhibiting cell growth	/	[79]
	Pancreatic cancer	Inducing ferroptosis	Regulating survival prediction-related genes	[80]
	Prostate cancer	Inhibiting cell growth and migration, inducing apoptosis	Reducing UHRF1 and inducing p16	[81]
	Rhabdomyosarcoma	Inhibiting cell growth	Inhibiting the mTORC1 pathway by inducing the AMPK pathway	[82]
Artesunate	Bladder cancer	Inducing cell death	Inducing ROS production and activating the AMPK-mTOR-ULK1 pathway	[83]
	Insulinoma	Inducing ferroptosis	Downregulating the SLC7A11/GPX4 pathway	[84]
	Lymphoma	Inhibiting cell growth, inducing G2/M cell cycle arrest and cell death	Inducing ROS production and TRF2 degradation	[85]
		Inducing apoptosis, autophagy, and ferroptosis	Inhibiting the E2F5/TFPI2/MMP pathway	[86]
	Renal cancer	Inducing cell growth and cell death, inhibiting anaerobic glycolysis	Inducing ROS production and regulating P53	[87]
	Thyroid cancer	Inhibiting cell growth, invasion, and migration	Inhibiting the PI3K/Akt/FKHR pathway	[88]
Anhydrodihydroartemisinin (10)	Prostate cancer	Inhibiting cell growth and migration	Modulating the caspase-dependent pathway	[89]

**Table 2:** Clinical trials of artemisinin and its derivatives against cancers.

Compound	Aim	Target	Design ^a^	Case/Control	Dose Regimen ^b^	Duration	Main Outcome Measures ^c^	Ref.
Artemisinin	Clinical effects and safety	Prostate cancer	CCT	15/-	p.o., 300–400 mg, t.i.d.	3 years	PSA doubling time, velocity, signs and symptoms of metastasis and survival	[109]
	Safety, tolerability, and pharmacokinetics	Advanced solid tumor malignancies	CCT	19/-	i.v., 8, 12, 18, 25, 34, and 45 mg/kg, once a week	21 days	MTD and DLTs, clinical activity as well as pharmacokinetic analysis	[110]
Artesunate	Clinical effects and safety	Colorectal cancer	RCT	12/11	p.o., 200 mg, q.d.	14 days	Apoptosis proportion of cancer cells, tumor marker expression (VEGF, EGFR, c-Myc, CD31, Ki67, and p53), and clinical responses	[111]
	Safety and tolerability	Metastatic breast cancer	CCT	23/-	p.o., 100, 150, 200 mg, q.d.	4 weeks	DL-AEs as well as neurological and audiological complications, ECG, full blood count including reticulocytes, ALAT, ASAT, creatinine, NTproBNP, and troponin T	[112]
			CCT	13/-	p.o., up to 200 mg, q.d.	Up to 37 months	AEs (clinical neurological examination, audiological examinations, ECG, hematology, clinical chemistry, CA 15-3, liver ultrasound)	[113]
			CCT	23/-	p.o., 100, 150, 200 mg, q.d.	4 weeks	DL-AEs as well as laboratory assessments, neurological, cardiological, and audiological examinations	[108]
	Pharmacokinetics	Metastatic breast cancer	CCT	23/-	p.o., 100, 150, 200 mg, q.d.	3 weeks	The population pharmacokinetic properties of artesunate and dihydroartemisinin in plasma	[12]
Artenimol-R	Clinical effects and safety	Advanced cervical cancer	CCT	10/-	p.o., 200 mg, q.d.	21 days	Pain and vaginal discharge symptoms, biomarker expression (p53, EGFR, Ki-67, CD31, and CD71) in biopsies	[114]

*^a^ RCT, randomized controlled trial; CCT, clinical controlled trial. ^b^ p.o., oral administration; t.i.d., three times a day; i.v., intravenous administration; q.d., once a day. ^c^ PSA, prostate specific antigen; MTD, maximum tolerated dose; DLTs, dose-limiting toxicities; DL-AEs, dose-limiting adverse events; ECG, changes in electrocardiogram; NTproBNP, B-type natriuretic peptide; AEs, adverse events.*
